# Angiotensin-Converting Enzyme (ACE) Inhibitors and Statins Mitigate Negative Cardiovascular and Pulmonary Effects of Particulate Matter in a Mouse Exposure Model

**DOI:** 10.3390/antiox15010106

**Published:** 2026-01-13

**Authors:** Tristan Junglas, Andreas Daiber, Ivana Kuntic, Arijan Valar, Jiayin Zheng, Matthias Oelze, Lea Strohm, Henning Ubbens, Omar Hahad, Maria Teresa Bayo Jimenez, Thomas Münzel, Marin Kuntic

**Affiliations:** 1 Molecular Cardiology, Department for Cardiology 1, University Medical Center Mainz, 55131 Mainz, Germanydaiber@uni-mainz.de (A.D.);; 2 German Center for Cardiovascular Research (DZHK), Partner Site Rhine-Main, 55131 Mainz, Germany; 3 Center for Thrombosis and Hemostasis (CTH), University Medical Center of the Johannes Gutenberg-University, 55131 Mainz, Germany

**Keywords:** particulate matter, oxidative stress, endothelial dysfunction, angiotensin-converting enzyme (ACE)-inhibitors, statins

## Abstract

Particulate matter (PM) is a significant contributor to air pollution-associated negative health effects, and cardiovascular disease patients are more susceptible to air pollution-mediated damage of the heart and vessels. The present study investigated the protective effects against PM-induced cardiovascular damage by classic cardiovascular drugs, as used for the standard therapy of cardiovascular disease patients. Male C57BL/6J mice were exposed to ambient PM_2.5_ (<2.5 µm) for 3 days with or without treatment with the cholesterol-lowering drug atorvastatin (20 mg/kg/d) or the angiotensin-converting enzyme (ACE) inhibitor captopril (50 mg/kg/d). Both drugs mitigated PM_2.5_-induced systolic blood pressure increases and partially prevented endothelial dysfunction, as reflected by a mixed effect on endothelial nitric oxide synthase phosphorylation. Both drugs ameliorated reactive oxygen species (ROS) formation and phagocytic nicotinamide adenine dinucleotide phosphate (NADPH) oxidase (NOX-2) expression in the vasculature of PM_2.5_-exposed mice. Pulmonary ROS levels showed a minor improvement by the treatments, whereas *Nox2* mRNA expression was not diminished. Only captopril showed some anti-inflammatory effects in the heart and lung of PM_2.5_-exposed mice, whereas both drugs failed to reduce systemic inflammation measured in plasma. These findings offer new insights into potential mitigation strategies for PM_2.5_-induced cardiovascular complications, particularly for patients at higher cardiovascular risk, like those with coronary artery or ischemic heart disease or hypertension.

## 1. Introduction

Advancements in medical care and hygiene have reduced the importance of communicable diseases for the global burden of disease, which is now dominated by non-communicable diseases, accounting for 74% of global deaths [1]. Cardiovascular diseases (CVDs) are responsible for 43% of all non-communicable disease-attributable deaths [1,2]. It is estimated that this share will further increase in the future [3]. Environmental and behavioral risk factors, such as noise, air, water, and soil pollution, and smoking, significantly contribute to the non-communicable disease burden [4]. Air pollution, particularly particulate matter (PM), has gained attention for its detrimental effects on human health [5,6]. Annual mortality rates attributable to air pollution range from 8.3 [7,8] to 10.2 million [9], accounting for almost 20% of all global deaths. In 2019, the Global Burden of Disease study ranked air pollution 4th among risk factors for disability-adjusted life years (DALYs), with only tobacco smoking, malnutrition, and high blood pressure ranking higher [2]. Particulate matter is generally classified by size into PM_10_ (<10 µm), PM_2.5_ (<2.5 µm), and ultrafine PM (<0.1 µm). As ultrafine PM is not commonly monitored, PM_2.5_ also encompasses particles of the ultrafine range. Major sources of PM_2.5_ are combustion (burning liquid fuels in engines and solid fuels for heating), tire and brake wear, and catalytic converter degradation, but also aggregation of gases on small mineral cores [10].

Early studies in laboratory animals have linked PM exposure to CVDs [11,12], with vascular oxidative stress and inflammation playing a significant role [13,14,15]. PM-induced oxidative stress causes endothelial dysfunction and arterial hypertension [16]. Also, oxidized lipids such as low-density lipoprotein (LDL) were observed upon PM exposure, promoting the progression of atherosclerotic plaques [17]. This raises questions about whether classic cardiovascular drugs like cholesterol-lowering statins and/or antihypertensive angiotensin-converting enzyme (ACE) inhibitors can protect against PM-induced cardiovascular dysfunction. Importantly, statins have shown beneficial effects on PM_2.5_-associated cardiovascular morbidity and mortality in epidemiological studies [18,19]. This has been attributed to their ability to reduce circulating LDL and their anti-inflammatory and antioxidant properties, resulting in protection against PM-induced oxidative stress [20]. In addition, statin therapy lowered C-reactive protein and other markers of inflammation in PM-exposed high-risk populations, such as diabetics, smokers, and the unmarried [21,22].

Another important cardiovascular drug group, the ACE inhibitors, was reported to protect against PM-induced hypertension [23] and endothelial dysfunction [24]. Captopril was among the selected medications that reversed genetic key markers modulated in human bronchial epithelial cells by ambient PM exposure [25]. Captopril also prevented urban particle-dependent activation of extracellular signal-regulated kinases 1 and 2 (ERK1/2) and p38 mitogen-activated protein kinases in human pulmonary artery endothelial cells [26]. However, not all studies support these protective actions of ACE inhibitors; e.g., a worsening of PM-induced changes in heart rate variability in response to captopril was observed [27].

Although the use of statin and ACE-inhibitor therapy in PM_2.5_ exposure was addressed previously in human studies, the mechanistic insight into the effects on endothelial dysfunction, oxidative stress, and inflammation is still lacking. The objective of this study was to provide a better mechanistic understanding of the pathological effects of PM_2.5_ exposure through pharmacological interventions with ACE inhibitors and statins.

## 2. Materials and Methods

### 2.1. Exposure of Laboratory Animals

Male C57BL/6 mice, aged 8–10 weeks, were used in this study. All mice were handled in accordance with the guidelines from Directive 2010/63/EU of the European Parliament on the protection of animals used for scientific purposes and were approved by the Ethics Committee of the University Medical Center Mainz and the Landesuntersuchungsamt Rheinland-Pfalz (Koblenz, Germany; permit number: 23 177-07/G 16-1-055). Prior to experimental sessions, all mice were held in a ventilated animal cabinet with a 12 h light/dark cycle and fed standard chow ad libitum. Experimental mice were divided into 4 groups: fresh air control, PM_2.5_-exposed group, PM_2.5_-exposed group with atorvastatin, and PM_2.5_-exposed group with captopril. PM_2.5_ exposure was performed in a custom exposure system (described in detail in Ref. [11]), built by TSE Systems GmbH (Hochtaunuskreis, Germany). A PM_2.5_ mixture was obtained from the National Institute of Standards and Technology (NIST) as a standard reference material (SRM1648a; a detailed certificate of analysis can be found at https://tsapps.nist.gov/srmext/certificates/1648a.pdf accessed on 11 January 2026). This PM_2.5_ preparation was collected from ambient air and is fully characterized for heavy metals and organic toxicants. The exposure was monitored by a custom particle detector (NanoSpectroPan, TSE Systems GmbH, Berlin, Germany). The exposure lasted for 6 h per day for 3 days. Atorvastatin (Biomol, Hamburg, Germany) and captopril (Merck, Darmstadt, Germany) treatment started one day before the PM_2.5_ exposure. Both drugs were administered via drinking water with the target doses of 20 mg/kg/d (atorvastatin [28]) and 50 mg/kg/d (captopril [29]). One day after the last exposure, mice were sacrificed under deep ketamine/xylazine anesthesia (i.p. 120/16 mg/kg body weight), and tissues were harvested for further analysis. The average concentration of urban particulate matter in the exposure chamber was 214 ± 65 µg/m^3^. The PM_2.5_ concentration range was selected based on 200–300 µg/m^3^, which is the peak concentration reached in the major polluted cities [30,31]. A mouse respiratory rate of 80–230 min^−1^ [32] and tidal volume of 0.2 mL [33], assuming a chamber concentration of 200 µg/m^3^, results in 1.15–3.31 µg exposure (assuming 100% retention). Mouse weight was 25 g, and the 6 h exposure session resulted in 46–132 µg/kg/day of PM_2.5_ exposure. A human respiratory rate of 10–20 min^−1^, tidal volume of 0.5 L, and body mass of 60 kg result in 24–48 µg/kg/day PM_2.5_ exposure. Assuming a 200 µg/m^3^ PM_2.5_ concentration. Since mouse exposure is performed during the sleeping phase when the respiratory activity is in the lower range, it is assumed that mouse and human exposures are similar.

**Figure 1:**
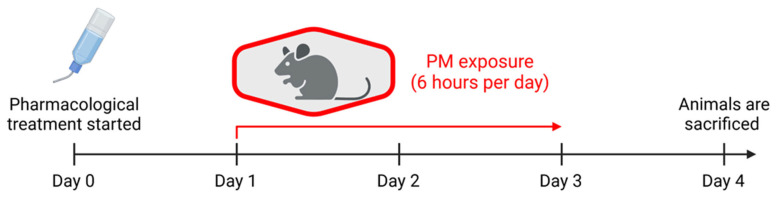
Mouse treatment and exposure paradigm. Created in BioRender. Kuntic, M. (2026) https://BioRender.com/10zhn7i (accessed on 6 November 2025).

The mouse exposure paradigm is shown in Figure 1.

### 2.2. Non-Invasive Blood Pressure Measurement

Blood pressure of exposed mice was measured by tail-cuff plethysmography using a CODA instrument (Kent Scientific, Torrington, CT, USA) (described previously [34,35]). During the measurement, mice were placed inside a plastic restrainer and positioned on a preheated plate (32 °C). The occlusion cuff and volume pressure recording cuff were placed on the tail of each mouse. Ten measurement iterations were performed for each mouse, and the mean value was reported. Mice were trained on at least two separate occasions before obtaining the baseline measurement. The final blood pressure was obtained directly after the last exposure to PM_2.5_ on the last day of exposure. All blood pressure measurements were performed during the same part of the day, including the training, to avoid circadian rhythm effects.

### 2.3. Isometric Tension Studies in Isolated Aortic Rings

After tissue harvest from the mice, a 4 mm segment of the thoracic aorta was cleaned of perivascular adipose tissue. This segment was then suspended in an organ bath chamber where force transducers were set up to measure the force applied by the vascular muscle cells. The procedure was described previously in detail [34,35]. Briefly, acetylcholine (ACh—endothelium-dependent vasodilator) was titrated (from 10^−9^ to 10^−5.5^ M) to the pre-constricted aortic segments (prostaglandin F_2α_ (Sigma Aldrich, Darmstadt, Germany)—yielding approximately 80% of the maximal force induced by KCl bolus), and the exerted force was recorded, yielding relaxation curves. During the measurement, the temperature was maintained at 37 °C, and carbogen gas (95% oxygen, 5% CO_2_ *v*/*v*) was constantly being bubbled. Prevention of unwanted prostaglandins and other vasoactive eicosanoids was achieved by introducing the cyclooxygenase inhibitor indomethacin (10 μM). The results are presented as relaxation curves and E_max_ and pEC_50_. From some mice, multiple rings were used.

### 2.4. Dihydroethidium Fluorescence Microtopography

A 3 mm segment of the thoracic aorta and a piece of the right lung were embedded in optimal cutting temperature (OCT) compound (TissueTek^TM^, Sakura Finetek, Umkirch, Germany). The procedure was described previously in detail [11,36]. In brief, the frozen OCT blocks were cut to a thickness of 8 µm and transferred onto SuperFrost^®^ (VWR International, Darmstadt, Germany) glass microscopy slides. Slides were then incubated with 1 µM dihydroethidium (DHE, Sigma Aldrich, Darmstadt, Germany) for 30 min at 37 °C to let the superoxide and H_2_O_2_ oxidize the dye. After the incubation, slides were washed 2 times with PBS, and a coverslip was applied to stop tissue drying. Fluorescent images were taken under a microscope (Axiovert 40CFL with Axiocam MRm, Zeiss, Jena, Germany). Optical parameters were set to excitation 510–520 nm and emission 580–610 nm (red light). The red fluorescence obtained in this way comes from both DHE oxidation products, 2-hydroxyethidium and ethidium, providing a measurement of total reactive oxygen species (ROS) production and not specifically superoxide or H_2_O_2_ individually [37,38]. The obtained images were quantified using mean pixel intensity in ImageJ software, version 1.52a.

### 2.5. Western Blot and Dot Blot Analysis

A standard Western blot analysis was performed to quantify the expression and/or phosphorylation of proteins of interest in aortic and pulmonary tissue [34,39]. After a gel electrophoresis and the transfer to a nitrocellulose membrane, proteins of interest were identified using specific primary antibodies: endothelial NO-synthase (eNOS, 1:1000, BD Bioscience #610297, San Jose, CA, USA), NADPH oxidase subunit NOX-2, mouse monoclonal, (1:500, BD Biosciences #611415, San Jose, CA, USA), heme oxygenase 1 (HO-1, 1:250, Abcam #ab68477, Cambridge, MA, USA), P-eNOS Thr495 (1:1000, Cell Signaling #9574S, Danvers, MA, USA), P-eNOS Ser1177 (1:1000 Cell Signaling, #9571, Danvers, MA, USA), and α-actinin (1:2500, Sigma-Aldrich #A5044, St. Louis, MO, USA) for normalization against loading and transfer. For the detection of phosphorylated eNOS, membranes were stripped and re-stained at the same kDa marker as eNOS. Secondary antibodies, conjugated to horseradish peroxidase (1:10,000 each, Vector Lab. #PI-2000 (anti-mouse IgG) and #PI-1000 (anti-rabbit IgG, Burlingame, CA, USA)) were used to visualize the proteins under an ECL Chemostar Imager (Intas Science Imaging Instruments GmbH, Göttingen, Germany).

The dot blot procedure was previously described in detail [34,35]. In brief, blood plasma was placed directly onto a nitrocellulose membrane under vacuum and dried at 65 °C for 60 min. After drying, the membrane was stained with Ponceau S (Sigma-Aldrich) for loading control. The membrane was then blocked and incubated with primary antibody for cluster of differentiation 68 (CD68, 1:1000, Abcam #ab31630, Cambridge, MA, USA). Secondary antibodies, conjugated to horseradish peroxidase (mentioned above), were used to visualize the proteins under an ECL Chemostar Imager. Both Western blot and dot blot data were quantified using the Gel-Pro Analyzer software, version 6.3.

### 2.6. Real-Time Quantitative PCR

Total mRNA was isolated from the whole frozen lung and heart tissue. Tissues were homogenized in a Tissue Lyser (QIAGEN, Hilden, Germany) using guanidine thiocyanate (GIT) buffer (4 M Guanidiniumisothiocyanat, 25 mM SodiumCitrat pH = 7.0, 0.5% N-Laurylsarcosine), and total mRNA was extracted using phenol (Roti aqua phenol #A980.2, Carl Roth, Karlsruhe, Germany)/chloroform/isoamyl alcohol (Roti–C/I #X984.1, Carl Roth, Karlsruhe, Germany). After centrifugation for 20 min at 14,000× *g* and 4 °C and separation of the aqueous phase, total mRNA was precipitated in 2-propanol at −20 °C overnight. The mRNA was centrifuged for 20 min at 14,000× *g* and 4 °C and washed with ice-cold 80% ethanol. After washing, RNA was dissolved in RNase-free water, and the total concentration was determined by a photometer (BioPhotomoeter, Eppendorf, Hamburg, Germany), and purity was checked by measuring the ratio of absorbance at 260 and 280 nm. Only the samples with a ratio of 1.90 or greater were used. 50 ng of total mRNA was used for quantitative reverse transcription real-time PCR (qRT-PCR) analysis using the QuantiTect Probe RT-PCR kit (QIAGEN, Hilden, Germany) [35]. The reaction mixture consisted of 10 µL of Master mix, 6.8 µL of RNase-free water, 1 µL of primer, and 0.2 µL of enzyme solution (part of the kit). The TaqMan^®^ dual-labeled fluorescence resonance energy transfer (FRET) primer-probe mixes were purchased from Applied Biosystems (Foster City, CA, USA) and used to analyze the mRNA expression. Expression of the following genes was measured: NOX-2 (NADPH oxidase 2–*cybb*-Mm00432775_m1), CD68 (cluster of differentiation 68-*cd68*-Mm00839636_g1), IL-6 (interleukin 6–*il6*-Mm00446190_m1), and the TBP (TATA box binding protein–*tbp*-Mm00446973_m1) as a loading control. Sequences of all primers can be accessed through the Thermo Fisher website based on the provided IDs, together with the reference genes. The PCR protocol was executed on the 7900HT Fast Real-Time PCR System (Applied Biosystems, Darmstadt, Germany). The protocol, using single-step reverse transcription, consisted of holding the step at 50 °C for 30 min and then at 95 °C for 15 min, followed by 40 cycles of 94 °C for 15 s and 60 °C for 1 min. For quantification of the relative mRNA expression, the comparative ΔΔCt method was used.

### 2.7. Statistics

Wherever possible, the results are presented as jitter plots, showing individual biological replicates. Statistical significance was calculated by either a one-way ANOVA or a 2-way ANOVA (for each ACh value in the relaxation curves) and using Tukey’s post hoc analysis for comparison of multiple means (correction using statistical hypothesis testing). All data sets passed at least one of the normality tests provided by GraphPad Prism (D’Agostino and Pearson test, Anderson–Darling test, Shapiro–Wilk test, and Kolmogorov–Smirnov test). Relaxation curves are shown with a nonlinear curve fit (sigmoidal) of the log(ACh) versus response, which was used to determine E_max_ and pEC_50_. All statistical analyses were performed in GraphPad Prism for Windows software, version 10. The number of replicates in the different assays may vary since not all animals were used in all assays. Due to the great demands on tissue from some methods, not all tissues were used for all experiments, resulting in a different number of samples in each experiment.

## 3. Results

**Figure 2:**
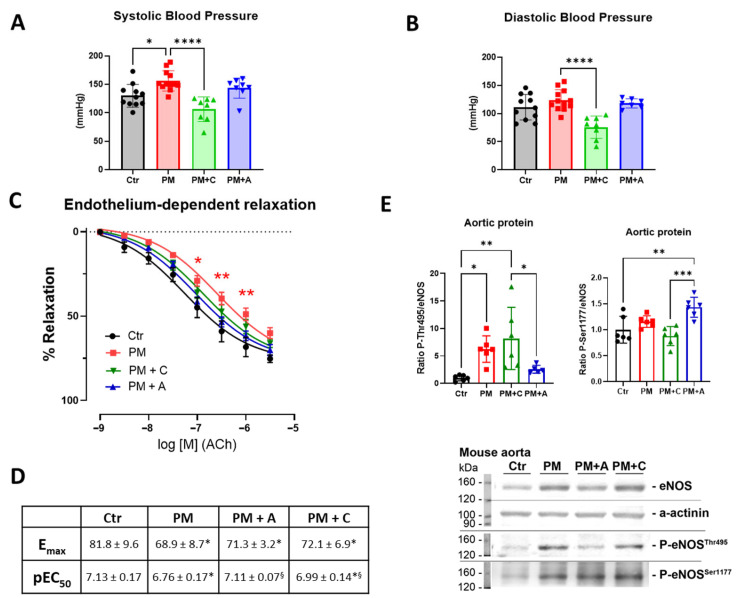
Blood pressure and vascular function. Systolic (**A**) and diastolic (**B**) blood pressure were measured by the non-invasive tail cuff method. Endothelium-dependent vascular relaxation of isolated aortic rings is presented as fitted relaxation curves (**C**) together with the E_max_ and pEC_50_ values (**D**). Ratios of phosphorylated endothelial nitric oxide synthase (P-eNOS) at Thr495 and Ser1177 against protein expression of eNOS are shown together with representative blots (**E**). Data are presented as mean ± SEM from 11 to 17 aortic rings originating from n = 8–12 mice per group (Ctr = 11; PM = 11; PM + A = 17; PM + C = 15) (**C**,**D**), or jitter plots show the mouse number for other parameters (n = 6–12 mice per group) in panels A, B, and E. Statistical significance is presented with * (*p* < 0.05), ** (*p* < 0.01), *** (*p* < 0.001), and **** (*p* < 0.0001) against the control (Ctr) and § (*p* < 0.05) against the PM group in table D.

Systolic blood pressure of PM_2.5_-exposed animals was increased, which was prevented by the treatment with either captopril or atorvastatin (Figure 2A). Diastolic blood pressure was not increased in the PM_2.5_ exposure group (Figure 2B). Treatment with the ACE inhibitor reduced both systolic and diastolic blood pressure below the control level. Endothelium-dependent vascular relaxation in response to acetylcholine (ACh) was impaired after exposure to PM_2.5_, and both treatments improved endothelial dysfunction (Figure 2C,D). Impairment of the endothelium-dependent vascular relaxation was also accompanied by an increase in the ratio of aortic phosphorylated eNOS at threonine 495 to non-phosphorylated eNOS (P-eNOS Thr495/eNOS) in the PM_2.5_-exposed group. There was no change observed in the ratio of aortic phosphorylated eNOS at serine 1177 to non-phosphorylated eNOS (P-eNOS Ser1177/eNOS) in the PM_2.5_-exposed group. Atorvastatin treatment increased the P-eNOS Ser1177/eNOS and mitigated the increase in P-eNOS Thr495/eNOS, pointing to the promotion of positive eNOS regulation (Figure 2E).

**Figure 3:**
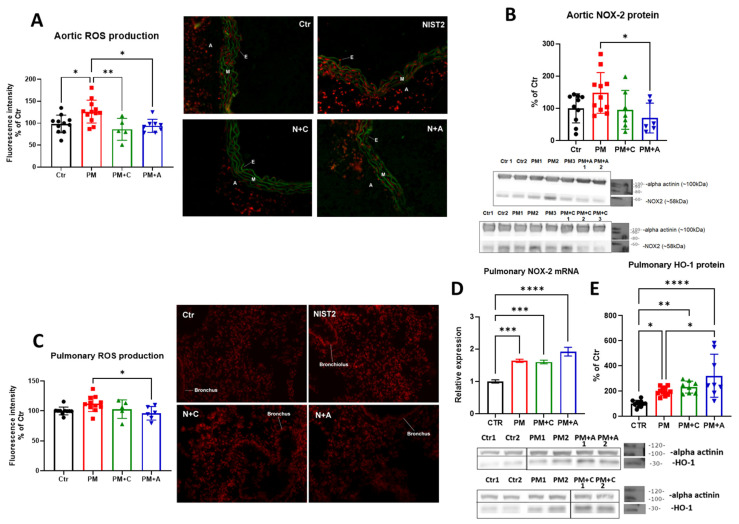
Cardiovascular and pulmonary oxidative stress. Dihydroethidium staining of the aortic sections is shown with representative images (**A**) (A—adventitia, M—media, E—endothelium). Aortic protein expression of NOX-2 (**B**) is shown with representative blots. Dihydroethidium staining of the lung sections is shown with representative images (**C**). Lung mRNA expression of *Nox2* (**D**) and protein expression of HO-1 (**E**) are shown with representative blots. The mouse numbers in panel D were n = 6 per group, and the mouse numbers for other panels are shown by jitter plots (n = 5–12 mice per group). Asterisks represent statistical significance: * (*p* < 0.05), ** (*p* < 0.01), *** (*p* < 0.001), and **** (*p* < 0.0001).

Vascular oxidative stress, as envisaged by the DHE staining of aortic sections, was significantly increased in the PM_2.5_ exposure group. The treatment with both captopril and atorvastatin ameliorated the effect (Figure 3A). The increase in oxidative stress was accompanied by an increase in aortic NOX-2 expression by trend (0.2 > *p* > 0.05), acting as a possible source of superoxide radicals, and both pharmacological treatments mitigated the NOX-2 expression and showed no significant increase (Figure 3B).

In addition to vascular oxidative stress, we also observed an increase in pulmonary oxidative stress after PM_2.5_ exposure by trend, which was completely ameliorated by atorvastatin treatment (Figure 3C). The mRNA expression of *Nox2* was increased, but no improvement was observed after the treatments with either of the drugs (Figure 3D). A similar result was observed in the protein expression of Nrf2-associated protein heme oxygenase 1 (Figure 3E), pointing to the activation of the antioxidant defense mechanisms.

**Figure 4:**
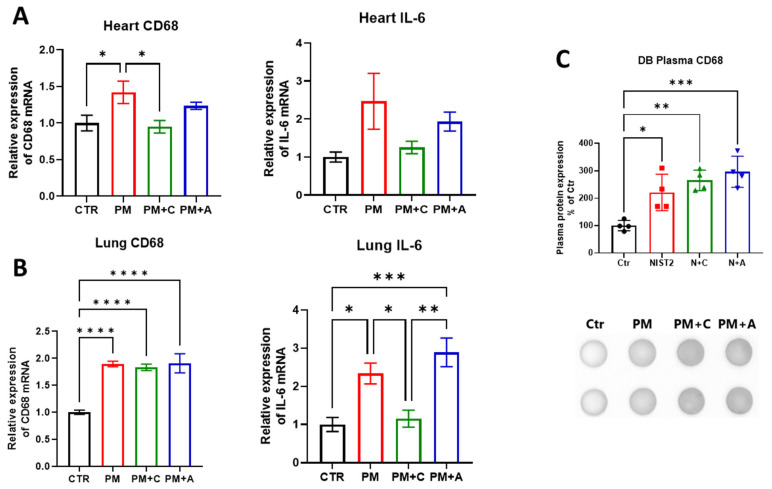
Systemic inflammation. Expression of mRNA for selected markers of inflammation, *Cd68*, and *Il6* was measured by RT-qPCR in cardiac (**A**) and pulmonary tissue (**B**). Circulating levels of CD68 were measured by the dot blot technique and were presented together with representative blots (**C**). The mouse numbers in panels A and B were n = 6–9 per group (Heart CD 68: n = 6; Heart IL-6: Ctr = 9; PM = 9; PM + A = 8; PM + C = 8; Lung CD68 Ctr = 9; PM = 9; PM + A = 8; PM + C = 8; Lung IL-6: n = 6), and the numbers in panel C are shown by jitter plots (n = 4 mice per group). Asterisks represent statistical significance: * (*p* < 0.05), ** (*p* < 0.01), *** (*p* < 0.001), and **** (*p* < 0.0001).

Exposure of mice to PM_2.5_ induced a systemic pro-inflammatory response. In the cardiac tissue, *Cd68* mRNA expression was increased after PM_2.5_ exposure and mitigated in both treatment groups. *Il6* also showed a clear trend toward increased expression in the PM_2.5_-exposed group and a trend toward mitigation with captopril treatment, while atorvastatin showed inconclusive results (Figure 4A). Pulmonary expression of *Cd68* was increased in the PM_2.5_-exposed group, as well as in both pharmacological treatment groups, while the PM_2.5_-induced increase in expression of *Il6* was mitigated by captopril, but not by atorvastatin treatment (Figure 4B). Circulating levels of CD68 were also increased with PM_2.5_ exposure, but no reduction was observed with either pharmacological treatment (Figure 4C).

## 4. Discussion

**Figure 5:**
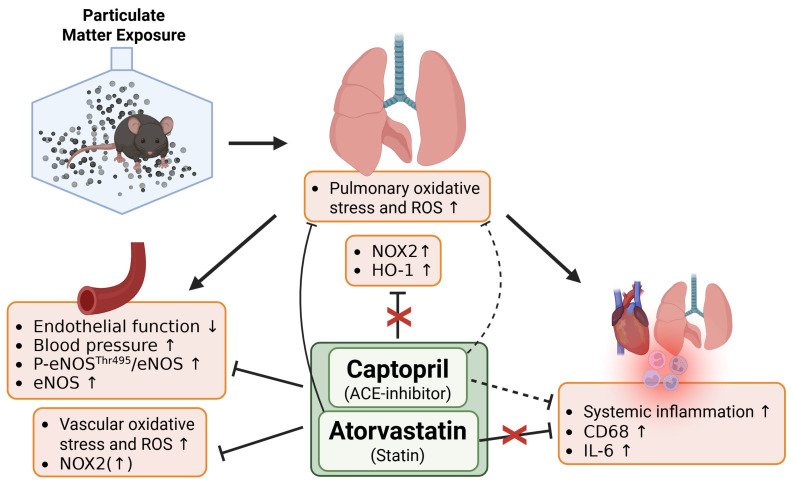
Conceptual summary of the obtained results. Both captopril and atorvastatin treatments were able to mitigate PM-induced endothelial dysfunction and vascular oxidative stress. Although both treatments reduced pulmonary ROS production, all other markers of oxidative stress remained elevated. Only captopril tended to reduce systemic inflammation caused by PM exposure, while atorvastatin treatment had no effect at all. Arrows pointing up or down indicate upregulation or downregulation respectively. Dashed lines represent uncertain or weak effects, while the solid lines represent a well established effect. Created in BioRender. Kuntic, M. (2026) https://BioRender.com/lu8fq7t (accessed on 11 January 2026).

The present study demonstrates that ACE inhibitor or statin administration can improve vascular function, oxidative stress, and inflammation in the vasculature and, to some extent, in the lungs in a mouse model of PM_2.5_ exposure. Both treatments positively affected endothelium-dependent vascular relaxation and partially normalized aortic eNOS expression and phosphorylation. However, pharmacological treatments exhibited varying effectiveness in mitigating PM_2.5_ effects on vascular and pulmonary oxidative stress and cardiac and pulmonary inflammation. This variation could be attributed to individual mechanisms of action. A summary of the results is presented in Figure 5.

### 4.1. Effects of PM_2.5_ on Vascular Function

Air pollution-derived PM has been found to negatively impact human vascular function, with studies confirming this through impaired flow-mediated dilation (a technique used to assess endothelial function in humans [40]) [41,42,43] and increased systolic blood pressure [44]. The proposed mechanisms for PM_2_._5_-induced end-organ damage include the translocation of smaller PM_2.5_ into circulation, which can cause physical or oxidative damage, or the induction of inflammation in the lung, which later becomes systemic and disrupts vascular signaling [16]. Smaller particles (mostly ultrafine particles with a diameter < 100 nm; potentially also fine PM_2.5_ with a diameter ≤ 2.5 µm) easily penetrate the lung and transmigrate into the circulation [45], whereas larger particles (coarse PM with a diameter ≤ 10 µm) rather become stuck and accumulate in the lung [46]. Animal studies have also observed impairment in vascular function and increased blood pressure due to PM exposure [11,47,48], thereby supporting the findings presented here. A head-to-head comparison of nanometer- versus micrometer-sized synthetic particles confirmed the higher potential of the ultrafine particles in causing cardiovascular damage and complications [49].

Our study reveals that eNOS expression increases with PM_2.5_ exposure, contradicting some literature findings suggesting a downregulation [50,51,52]. However, some studies show an increase in eNOS expression due to shorter exposure time [53,54], where compensatory transcription and translation of eNOS are activated to mitigate the lack of nitric oxide production, a concept proposed by us previously [55,56]. In addition, we have several times demonstrated that in the setting of increased oxidative stress in the endothelium, eNOS is upregulated, e.g., in the setting of angiotensin II infusion [57], diabetes mellitus [58], and noise exposure [34]. This upregulation is likely mediated by H_2_O_2_, which has been shown to upregulate eNOS at the transcriptional level [59]. In contrast, chronic exposure to PM_2.5_ has been shown to result in a downregulation of eNOS [60,61]. We also show here that the PM_2.5_ exposure increased the ratio of P-eNOS Thr495 to eNOS but not the ratio of P-eNOS Ser1177 to eNOS. Phosphorylation of eNOS at Thr495 is considered negative, and phosphorylation at Ser1177 is considered positive for ·NO production [62]. This observation confirms that eNOS is negatively regulated in the PM_2.5_-exposed mice and that the increase in total eNOS expression is a result of compensatory mechanisms trying to reestablish the ·NO homeostasis. For a full understanding of eNOS uncoupling, more detailed measurements would be needed, including BH_4_ redox balance and ·NO bioavailability.

The treatment with ACE-inhibitor captopril lowers blood pressure, likely due to an improvement of endothelial dysfunction due to a reduction in oxidative stress [63]. ACE inhibition with captopril has been demonstrated to correct PM-induced adverse vascular reactivity due to passive smoking in the rabbit vasculature [64]. Captopril also normalized hypertension caused by the environmental stressor, aircraft noise [29]. All these data explain at least in part the above-mentioned association of arterial hypertension in humans caused by PM_2.5_ and the beneficial effects of ACE inhibitors on endothelial dysfunction [23,24]. Likewise, the normalization of altered gene expression related to inflammation, ferroptosis, autophagy, and MAP kinase activity in PM-treated cultured cells by captopril could provide the basis for the antihypertensive and vasoprotective effects of the drug in our model and clinical studies [25,26].

The beneficial effect of atorvastatin on endothelial function in PM_2.5_-exposed mice can be attributed to the well-known antioxidant and anti-inflammatory effects of statins [65,66], although the latter was not observed in the present study. Atorvastatin has been shown to prevent endothelial dysfunction, vascular oxidative stress by nicotinamide adenine dinucleotide phosphate (NADPH) oxidases and uncoupled eNOS, and compensatory upregulation of eNOS in a rat model of diabetes [28]. These data support the above-mentioned human data of the beneficial effects of statins on PM_2.5_-induced cardiovascular morbidity and mortality and may provide an explanation for the vasoprotective actions of cholesterol-lowering medications [18,19]. Likewise, the normalization of higher LDL levels along with a reduction in inflammation and oxidative stress in PM-treated rabbits and cultured cells by statins explains the pleiotropic, antioxidant, and vasoprotective effects of these drugs in our model and clinical studies [20,67]. As a proof-of-concept, populations with a higher cardiovascular risk without statin medication showed more pronounced inflammation by PM exposure than those taking statins [21,22,68,69,70,71]. Here, we show that atorvastatin mitigates the negative effects of PM exposure on eNOS by lowering the P-eNOS Thr495 to eNOS and increasing the P-eNOS Ser1177 to eNOS ratio.

### 4.2. Effects of PM on Oxidative Stress and Inflammation

Oxidative stress is a hallmark of most chronic diseases [72], especially CVDs [73], and was identified as a central pathomechanism of PM exposure [74,75]. PM_2.5_, a common air pollution-derived toxicant, can cause oxidative stress in the lung, which is the first point of impact for any PM material [76]. The oxidative stress can originate from ROS present on the PM itself or endogenously from ROS-generating enzymatic processes (based on, e.g., NOX-2, uncoupled eNOS, or mitochondria) induced by the presence of PM [77,78]. A central role of inflammatory cells and phagocytic NADPH oxidase for PM_2.5_-mediated cardiovascular damage and complications was previously shown, also by using knockout mice as a proof-of-concept [79]. Oxidative stress is a major trigger of endothelial dysfunction in PM-exposed humans and animals [80,81]. In this study, PM_2.5_-induced increases in mRNA and protein levels of NADPH oxidase complex constituent NOX-2 in the lung and aorta were observed. Neither pharmacological treatment with the blood pressure-lowering drug nor the cholesterol-lowering medication alleviated the expression of NADPH oxidase in the lung, but statin treatment lowered total oxidative stress. In the aorta, both drugs suppressed ROS formation and diminished NOX-2 expression levels. The minor effect of PM_2.5_ exposure on pulmonary ROS might be due to the high background of the DHE oxidation product fluorescence. The antioxidant defense protein heme oxygenase 1 (HO-1) represents a counter-regulatory response to increased ROS formation by oxidative activation of the transcription factor Nrf2, which is observed in almost any chronic disease [82]. In the present study, HO-1 expression was upregulated by PM_2.5_ exposure in the lung, which was, however, not corrected by the drugs. For cardiovascular oxidative stress, both pharmacological interventions generally have a positive effect, reducing total ROS production in the aorta and alleviating the PM_2.5_-induced elevation in aortic NADPH oxidase complex constituents. NADPH oxidase-derived ROS can also influence eNOS function through uncoupling, leading to endothelial dysfunction in PM_2.5_-exposed animals [11,83]. Other mechanisms could also explain the increase in total ROS production after PM_2.5_ exposure and mitigation by drug treatments, such as mitochondrial dysfunction [84].

Inflammation is closely linked to oxidative stress, as it is a source of ROS and is driven by ROS [85]. PM exposure can induce systemic inflammation, with elevated levels of inflammation markers in the lung, heart, and circulation [86,87,88]. This was confirmed in PM-exposed mice by the pro-inflammatory phenotype of the vessels, promotion of atherosclerosis [89], and the involvement of the Toll-like receptor 4 and the NLRP3 inflammasome in these processes [79,90]. Here, treatments with ACE inhibitors or statins showed improvement in PM-induced cardiac inflammation by trend, but only partially improved pulmonary inflammation markers or failed to ameliorate circulating inflammatory mediators. Pulmonary inflammation is expected to remain high, as accumulating PM in the lung remains a potent antigen for resident macrophages and can gain a systemic character through the release of pro-inflammatory cytokines. This was observed even after cessation of PM exposure [91] and suggests that circulating cytokines may remain elevated also after pharmacological intervention. An increase in plasma CD68 might not fully represent systemic inflammation, and future studies should examine a broader panel of inflammation markers. The general lack of effects of both drugs in mitigating pulmonary NOX2 expression and antioxidant response via HO-1 and inflammation could be due to the accumulation of particles in the lungs, providing an additive stimulus for the immune system due to the long removal process [92].

### 4.3. Importance for Vulnerable Groups

Chemical pollution, in general, has a large impact on premature deaths and DALYs in vulnerable groups such as children and the elderly [93,94]. Air pollution, in particular, has a significant impact on vulnerable groups, including preexisting cardiovascular disease patients [95,96,97]. Therefore, classic cardiovascular therapies are being investigated to alleviate the health burden associated with PM_2_._5_ exposure. The present study found that pharmacological interventions of ACE inhibitors and statins can improve PM_2.5_ exposure-induced vascular function and oxidative stress. ACE inhibitors decrease blood pressure, while both drugs have antioxidant properties that improve vascular functional parameters. However, these interventions only confer partial reduction in pulmonary oxidative stress or systemic inflammation, suggesting other mechanisms could worsen preexisting cardiovascular disease in chronic exposure scenarios. The ability of classic cardiovascular drugs to mitigate acute PM_2.5_ exposure-derived endothelial dysfunction provides a positive outlook for areas with seasonal or rare event-associated increases in PM_2.5_ concentrations.

### 4.4. Limitations of the Study

In the present study, both drugs were administered one day before and during the PM_2_._5_ exposure. Administration of drugs before the negative effects of the treatment appear is not the usual way in which humans are medicated, but it provides a mechanistic insight into the importance of the modulated pathway. On the other hand, patients who are already taking chronic therapies might experience acute exposures to PM_2.5_, making our exposure protocol a valid real-life scenario. The high dose of PM_2.5_ and the acute exposure protocol do not necessarily provide a good model for chronic exposure and long-term risk, although some studies show that high peak PM concentrations seem to trigger ischemic events such as acute myocardial infarction (AMI) [98,99]. We used only male mice due to the impact of female sex hormones, such as estrogen, on vascular function [100], which is in line with our own observations on exacerbated vascular damage and dysfunction by various risk factors in male mice.

## 5. Conclusions

The aging population is increasing the number of people suffering from chronic cardiovascular diseases, making them more vulnerable to air pollution-derived PM. Classical cardiovascular drugs like ACE inhibitors and statins may provide protection against PM-associated effects in patients already taking this therapy. This represents a benefit for patients with CVDs who are already treated with standard cardiovascular medications. This study provides mechanistic insights into how these drugs mitigate the negative vascular effects of acute PM exposure, but more data are needed to fully understand the mechanisms involved. However, the here and elsewhere reported protective effects of ACE inhibitors and statins against PM_2_._5_-mediated cardiovascular damage cannot replace political mitigation strategies by lowering the legal limits of air pollution constituents.

## Data Availability

The original contributions presented in this study are included in the article. Further inquiries can be directed to the corresponding author.
